# Assessment of *Bona Fide* sRNAs in *Staphylococcus aureus*

**DOI:** 10.3389/fmicb.2018.00228

**Published:** 2018-02-20

**Authors:** Wenfeng Liu, Tatiana Rochat, Claire Toffano-Nioche, Thao Nguyen Le Lam, Philippe Bouloc, Claire Morvan

**Affiliations:** ^1^Institute for Integrative Biology of the Cell (I2BC), CEA, Centre National de la Recherche Scientifique, Université Paris-Sud, Université Paris-Saclay, Gif-sur-Yvette, France; ^2^VIM, Institut National de la Recherche Agronomique, Université Paris-Saclay, Institut National de la Recherche Agronomique Centre Jouy-en-Josas, Jouy-en-Josas, France

**Keywords:** *bona fide* sRNA, *Staphylococcus aureus* HG003, RNA-seq, transcription factors, gene regulation

## Abstract

Bacterial regulatory RNAs have been extensively studied for over a decade, and are progressively being integrated into the complex genetic regulatory network. Transcriptomic arrays, recent deep-sequencing data and bioinformatics suggest that bacterial genomes produce hundreds of regulatory RNAs. However, while some have been authenticated, the existence of the others varies according to strains and growth conditions, and their detection fluctuates with the methodologies used for data acquisition and interpretation. For example, several small RNA (sRNA) candidates are now known to be parts of UTR transcripts. Accurate annotation of regulatory RNAs is a complex task essential for molecular and functional studies. We defined *bona fide* sRNAs as those that (i) likely act in *trans* and (ii) are not expressed from the opposite strand of a coding gene. Using published data and our own RNA-seq data, we reviewed hundreds of *Staphylococcus aureus* putative regulatory RNAs using the DETR'PROK computational pipeline and visual inspection of expression data, addressing the question of which transcriptional signals correspond to sRNAs. We conclude that the model strain HG003, a NCTC8325 derivative commonly used for *S. aureus* genetic regulation studies, has only about 50 *bona fide* sRNAs, indicating that these RNAs are less numerous than commonly stated. Among them, about half are associated to the *S. aureus* sp. core genome and a quarter are possibly expressed in other *Staphylococci*. We hypothesize on their features and regulation using bioinformatic approaches.

## Introduction

Bacterial regulatory RNAs are essential elements of complex genetic networks that tune gene expression according to growth conditions (Wagner and Romby, 2015). Most of them associate by base pairing to target sequences, and affect stability, structure and translation efficiency of target RNAs. Regulatory RNAs are divided into two categories, *cis*- and *trans*-acting RNAs. *Cis*-acting RNAs regulate expression of adjacent genes without reaching their substrate by diffusion (Mellin and Cossart, 2015). In contrast, *trans*-acting RNAs are expressed from loci not necessarily genetically linked to their target. RNAs and in some cases proteins are targets of *trans*-acting RNAs. When a *trans*-acting RNA is expressed from a complementary strand of another gene, it is often called antisense RNA (asRNA) (Georg and Hess, 2011); the predicted target of these asRNAs is the RNA transcribed from the complementary sequence. *Trans*-acting RNAs that are not asRNAs are often referred to as sRNAs because most of them are of small size. In bacteria, they are usually 50–300 nucleotides long, non-coding and conditionally expressed (i.e., depending upon specific stress and/or growth phase), although several sRNAs do not fit this description. Of interest, RNAIII, which is over 500 nucleotides long and encodes delta haemolysin, is an “exceptional” staphylococcal sRNA paradigm (Novick et al., 1993). This example alone underlines the difficulty of giving a straightforward definition of a *bona fide* sRNA.

Since 2005, *S. aureus* non-coding RNAs have been searched by bioinformatics (Pichon and Felden, 2005; Geissmann et al., 2009; Marchais et al., 2009), DNA-arrays (Anderson et al., 2006; Roberts et al., 2006; Mäder et al., 2016), cDNA sequencing (Abu-Qatouseh et al., 2007, 2010), and RNA-seq methods (Beaume et al., 2010; Bohn et al., 2010; Howden et al., 2013; Broach et al., 2016; Carroll et al., 2016). The data are difficult to compare because of the different strains, growth conditions and experimental procedures used. In addition, many regulatory RNAs were renamed and in some cases, previously published work was overlooked. Data from different studies suggest that *S. aureus* may have hundreds sRNAs, but <10 have thus far been functionally characterized.

Despite recent releases of compilation and cross-comparison of available data in different *S. aureus* strains, (Felden et al., 2011; Sassi et al., 2015; Carroll et al., 2016; Mäder et al., 2016), it is still difficult to determine *bona fide* sRNAs from transcriptional background noise, asRNAs, and *u*n*t*ranslated *r*egion (UTR) derived RNAs. We applied rigorous criteria to define sRNAs, and then used visual curation and bioinformatic approaches on compiled experimental data to assess *bona fide* sRNAs in *S. aureus*. *S. aureus* HG003 (Herbert et al., 2010), an NCTC8325 derivative, was used as the model strain to list *bona fide* sRNAs. Our main objectives were to identify sRNAs likely to act in *trans* and to clarify redundancies in the literature due to the use of different nomenclature. We then performed *in silico* analysis on these sRNAs to determine their phylogenetic conservation and to predict their putative regulators. The reassessment of the number of expressed sRNAs in *S. aureus* provided by this study may be applicable to other bacteria.

### *Bona fide* sRNA definition

Bacterial genomes have complex organization with condensed information and flexible gene expression driven by multiple promoters with some internal to ORFs, operon organization, alternative premature termination, leader-less translation, and translational coupling (e.g., Mäder et al., 2016). The extent to which antisense RNA impacts gene expression in *S. aureus* is debatable, with reports of both high (Lasa et al., 2011) or more marginal (Mäder et al., 2016) effects. For these reasons, RNA boundaries are difficult to predict and may vary with strains and growth conditions.

**Figure 1:**
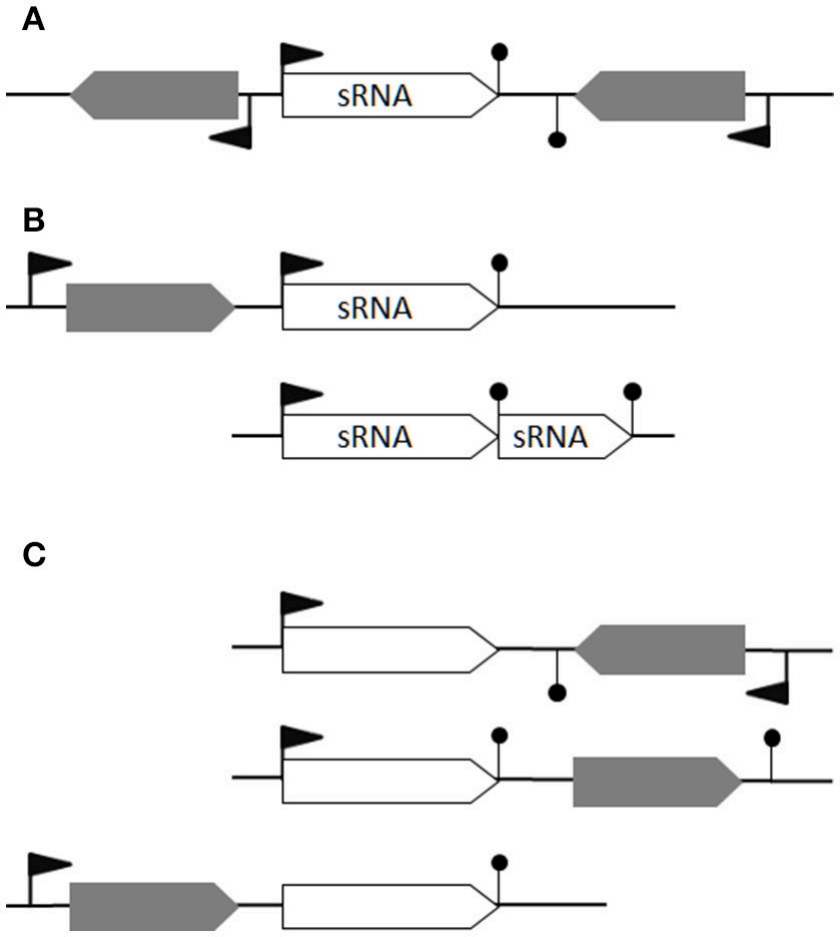
Defining *bona fide* sRNAs. **(A)** An ideal *bona fide* sRNA gene has its own promotor and transcriptional terminator. Its transcription does not overlap any antisense transcription. **(B)** In the first case, transcription from the second promoter leads to a *bona fide* sRNA while the transcription from the first promoter would likely generate a transcript with a long 3′UTR (e.g., RsaG). In the second case, a transcriptional termination read-through generates an alternative longer sRNA (e.g., RsaE-S390). **(C)** Three examples of non-coding RNAs not considered as *bona fide* sRNAs: a putative asRNA, a *cis*-regulatory element and a long 3′-UTR, respectively. Flag: promotor; hairpin loop: terminator; gray arrow: open reading frames; empty arrow: non-coding RNA.

We consider that a theoretical *bona fide* sRNA is (i) a gene not overlapping any other genes from the opposite strand, a definition excluding asRNAs, (ii) not a putative processed UTR and (iii) not a transcript derived from premature termination (i.e., riboswitch). It would therefore have its own promoter and a transcriptional terminator detected by computational predictions (Figure 1A), or interpreted as such because of clear expression up- and down-shifts (Figure 1B). This restrictive definition excludes processed UTRs and short transcripts from premature transcription termination that could also act in *trans* (Loh et al., 2009); riboswitches and long UTRs are thus excluded as putative sRNAs (Figure 1C). Type I toxin-antitoxin systems comprising a small open reading frame post-transcriptionally controlled by an antisense RNA are also excluded; this concerns several *spr* genes located within pathogenicity islands (Pichon and Felden, 2005).

The first RNA-seq studies were performed with low read densities. Reads distant from coding sequences and not homologous to known non-coding RNAs (e.g., tRNAs, rRNAs, known UTRs) were first interpreted as putative *bona fide* sRNAs, and consequently, compiling results from different publications on *S. aureus* overestimated the number of sRNAs *per* bacterial strain. Indeed, recent high-density RNA-seq and tiling-array data reveal that many sequences previously considered as sRNAs are UTRs or premature termination products from longer transcripts.

Another consequence of sRNA identification with low read coverage was ambiguous identification of transcription start and termination sites. Even for well-studied sRNAs (e.g., RsaE), transcript boundaries differ according to studies, possibly because of strains, and growth conditions used for experiments. The recent high density transcriptome information is used here to define sRNA boundaries.

### *Staphylococcus aureus* regulatory RNA data

*Staphylococcus aureus* genomes differ by the presence of variable elements (e.g., pathogenicity islands, SCCmec elements, prophages, transposable elements, insertion sequences, and plasmids). A recent study based on 64 *S. aureus* strains from different ecological niches reveals a core genome of 1,441 genes (not counting sRNAs genes, except tmRNA) and a pangenome of more than 7,400 genes, indicating a wide genetic diversity between strains (Bosi et al., 2016). Transcriptional patterns are influenced by these variable elements, which may affect virulence and antibiotic susceptibility. In addition, strain-specific elements have sRNA genes (Pichon and Felden, 2005) that can directly modulate core genome gene expression (Chabelskaya et al., 2010). sRNA expression has been experimentally studied by global approaches in different *S. aureus* strains, including NCTC8325 derivatives and methicillin resistant strains isolated in Japan, the USA and Europe (N315, USA300, MRSA252, respectively) (Felden et al., 2011; Sassi et al., 2015; Carroll et al., 2016; Mäder et al., 2016).

NCTC8325 (aka RN1) is a *S. aureus* strain isolated from a sepsis patient in 1960 widely used for genetic and physiological studies (Novick and Richmond, 1965). This strain is defective for two main regulators encoded by *rsbU* and *tcaR*: a positive activator of the general stress response regulator σ^B^ and a transcriptional activator of protein A-encoding gene, respectively. HG001, the strain used in the impressive transcriptional landscape study of 44 growth conditions published by Mäder et al. (2016), is a NCTC8325 derivative repaired solely for *rsbU*, whereas HG003, the strain we focus on in this analysis and that is now used as a model strain for *S. aureus* regulation studies (Herbert et al., 2010), is repaired for both *rsbU* and *tcaR* genes.

According to the compilation of data from several publications, *S. aureus* N315, NCTC8325, and Newman strains each could have over 500 putative regulatory RNAs (Sassi et al., 2015), the precise figure changing according to strains and sources (Carroll et al., 2016; Mäder et al., 2016). Most sRNAs were rediscovered in each independent analysis often under a different name. In order to compile an accurate list of *bona fide* sRNAs, we visually analyzed high-density coverage published data plus our own RNA-seq data (deep sequencing of pooled RNA extracts from cultures of HG003 strain grown in 16 different growth conditions; GEO GSE104971) as reported in Methods, and performed in-depth curation according to the rules defined in the previous chapter.

## Methods

### RNA-seq for sRNA detection

Experiments were performed with the *S. aureus* HG003 strain grown in different conditions: (i) eight samples in rich medium (BHI) at OD_600nm_ 0.6, 1.8, 3.3, 4.5, 7.2, 9.8, and 12.8, and stationary phase (24 h), (ii) seven samples under stress conditions (cold shock, heat shock, oxygen limitation, alkaline stress, oxidative stress, disulfide stress, iron-depleted condition and (iii) one sample from colonies on BHI-agar plates (also see GSE10497 in GEO database). Total RNAs were extracted from these 16 growth conditions, pooled together and processed using the MICROBExpress kit (Ambion, AM1905) as recommended by the suppliers, to remove rRNAs. They were then sequenced using an Illumina Genome Analyzer IIx generating single-end 40-nt reads. After a FastQC (v0.10.1) quality control, reads from the stranded and single-end sequencing were mapped onto the reference genome (*S. aureus* subsp. aureus NCTC8325, CP000253.1 version) using Bowtie 2 with default parameters and an overlapping rate of 69%. DETR'PROK_2.1.2.sh pipeline was run to detect sRNAs (Toffano-Nioche et al., 2012, 2013); parameters are supplied as supplementary materials. The logarithm of the read coverage was computed for multiple or unique mapping reads on each strand; values were used to identify RNAs containing repeated regions (home-made shell scripts) and visualized with the Artemis genome viewer (Rutherford et al., 2000).

### Literature and experimental data integration

*S. aureus* global studies available in literature are summed up in Table S1. sRNA annotations (coordinates and strand) were collected from the following whole transcriptome analyses: 255 “indep” (transcripts with a promoter determined independently of annotated features) or “inter” (between two annotated regions transcribed from independent promoters) (Mäder et al., 2016), 286 sRNAs (Carroll et al., 2016), 352 NCTC8325 automatically annotated sRNAs (Sassi et al., 2015) using HG003 RNA-seq data (this work; GEO GSE104971), and 53 sRNAs (Beaume et al., 2010). These sRNA annotations were pooled together as a GFF file for the present expert analysis. sRNA expression profiles and reported annotations from different strains were compared with HG001 transcription profiles of *S. aureus* expression data browser (http://genome.jouy.inra.fr/cgi-bin/aeb/index.py). This manual expertise led us to draw up a *bona fide* sRNA list with their most probable positions as described in Tables 1, 2.

**Table 1:** Bona *fide* sRNAs expressed in HG003.

**Name**	**Other names**	**Start**	**End**	**Strand**	**UCCC**	**Validations**	**Comments**
srn_0335	Includes SAOUHSCs258, S35	115205	115614	–	+	NB^a^^,^^b^	Repeated region
RsaG	Teg93, sRNA31, srn_0510, SAOUHSCs054, S58	201738	201962	+	+	NB^c^ RT^c^ 5′^c^	Own promoter + read-through from SAOUHSC_00183
Sau-5971	srn_0880, SAOUHSCs073, S109	361904	362002	–	–	NB^d^	
ptsrn_0890	sRNA71, SAOUHSCs205, SAOUHSC_A00354	367121	367211	–	–		Part of putative ORF SAOUHSC_A00354
Teg147	sRNA85, srn_0960, SAOUHSCs103	386294	386353	+	–		
ptsrn_1505	SAOUHSCs189, S204	569615	569939	+	–	NB^a^	Own promoter and 3'UTR from SAOUHSC_00559
RsaA	Teg88, Sau-64, sRNA132, srn_1510, SAOUHSCs048, S210	575845	575987	+	+	NB^c^^,^^d^ RT^c^ 5′^c^^,^^e^ 3′^e^	Sigma B regulation
ptRsaA_L_	*c.f*. above + RsaA-Sau-76, srn_1520, SAOUHSCs164, S211	575845	576126	+	+	NB^f^	Sigma B regulation Processed into two sRNAs
RsaC	Teg90, sRNA135, srn_1590, SAOUHSCs050, S234	623360	624458	–	+	NB^c^ 5′3′^c^	Internal repeat. Own promoter and read-through from SAOUHSC_00634
ptRsaD	sRNA138, srn_1640, SAOUHSCs051, S243	639711	639872	–	+	NB^c^ RT^c^	Antisense of putative SAOUHSC_00650 Antisense expression in some conditions
RsaH	Teg94, Sau-6059, sRNA162, srn_1910, SAOUHSCs055, S317	774294	774421	+	+	NB^c^ RT^c^ 5′ 3′^c^^,^^e^	Antisense of SAOUHSC_00792 promoter
pttmRNA	Teg150, ssrA, SAOUHSCs006, WAN014GIY, sRNA166, S329	788284	788675	+	+	NB^g^	Own promoter and read-through from SAOUHSC_00804
RsaE	Sau-20, Teg92, sRNA183, srn_2130, S389	911380	911481	+	+	NB^c^ RT^c^ 5′^c^^,^^e^ 3′^e^	Own promoter and read-through from SAOUHSC_00937
ptRsaE-S390	srn_2130, S389 + S390, includes RsaF	911380	911739	+	+		Poor expression; long product from RsaE terminator read-through
sRNA195	sRNA195, srn_2320, SAOUHSCs226, S414	990586	990684	–	–		Possible antisense of SAOUHSC_01018 3'UTR
RNA207	srn_2500, SAOUHSCs229	1078428	1078718	–	+		Internal repeat
Teg106	srn_2730, SAOUHSCs093, S540	1247774	1247925	+	+		Poor expression
ptTeg108	sRNA222, srn_2740, SAOUHSCs094	1248013	1248138	–	+		
srn_2975	SAOUHSCs275, S596	1362893	1363064	+	+	NB^a^^,^^b^	5' partly antisense of SAOUHSC_01422. Longer transcript with terminator read-through antisense of SAOUHSC_01423
S627	None	1462734	1462962	–	–		Own promoter and 3'UTR from SAOUHSC_01514. Repeated region. Antisense expression in some conditions
SprX2	Ssr6, RsaOR, Teg15, srn_3820.1, SAOUHSC_A01455	1464058	1464207	–	+	NB^e^^,^^h^ 5′^e^	Repeated region; putative ORF SAOUHSC_A01455; Possibly associated with S629
629	None	1464252	1464380	–	–		Possibly 5'UTR of SAOUHSC_A01455
6S RNA	Teg97, SsrS, Ssr80, WAN01CC8T, sRNA256, SAOUHSCs026, S685	1639003	1639243	–	–	NB^g^	Terminator read-through to SAOUHSC_01736
RNA264	srn_3320, SAOUHSCs017, S706	1685428	1685667	–	–		Terminator read-through to SAOUHSC_01787
srn_3355	SAOUHSCs110, included in S713	1707679	1707781	–	–		
Sau-5949	Teg120, sRNA272, srn_3460, SAOUHSCs070	1771663	1771728	+	–	NB^d^	Possible antisense of SAOUHSC_01865 3'UTR
srn_3555	SAOUHSCs221	1821336	1821444	+	–		Repeated region
SprB	Teg9, srn_3600, SAOUHSCs030	1849001	1849117	–	–	NB^g^^,^^d^	Not detected in Mäder et al.
sRNA287	srn_9340, SAOUHSCs236, S774	1863800	1863899	–	–		Own promoter + possible terminator read-through from SAOUHSC_T00050
srn_9345	S808	1923614	1923879	–	–	NB^a^	Own promoter + 3'UTR of SAOUHSC_02016
S810	None	1924486	1924611	–	–		Own promoter inside SAOUHSC_02019. Repeated regions.
ptTeg122	srn_3770, SAOUHSCs097	2027317	2027376	+	–		52 pb; in proximity of a putative type I TA system.
SprD	Teg14, sRNA300, srn_3800, SAOUHSCs032, S853	2033619	2033763	–	+	NB^g^^,^^d^	
ptTeg124	srn_3810	2033838	2033899	–	–		Not detected in Mäder et al.
SprX1	ssr6, RsaOR, Teg15, sRNA299, srn_3820, SAOUHSCs033, S854	2035228	2035378	–	+	NB^e^^,^^h^ 5′^e^	Repeated regions; possible 5'UTR of SAOUHSC_02170
ptRNAIII	sRNA317, srn_3910, SAOUHSCs022, S871	2093158	2093673	–	+	NB^g^^,^^b^^,^^d^ 5′3′^i^	SAOUHSC_02260 (*hld*) mRNA
RsaOG	RsaI, Teg24, sRNA356, srn_4390, SAOUHSCs047, S999	2377317	2377465	–	–	NB^b^^,^^c^^,^^j^ RT^c^^,^^k^ 5′c	Antisense expression in some conditions in Mäder et al. study.
Ssr42	RsaX28, Teg27, sRNA363, srn_4470, SAOUHSCs084, S1036	2446923	2448156	–	+		1252 pb; high constitutive transcription; terminator read-through antisense of SAOUHSC_02663
RsaX20	Teg128 + Teg130, srn_4520, SAOUHSCs100 (included), SAOUHSC_02702 + S1052	2484471	2484732	+	+		Contains putative ORF SAOUHSC_02702
Sau-19	Teg131, RsaX21, sRNA382, srn_4680, SAOUHSCs060	2556335	2556412	+	–	NB^d^	Not detected in Mäder et al.
Teg33	sRNA400, srn_5010, S1164	2721121	2721350	–	+		Own promoter; 3′UTR from SAOUHSC_02961; antisense of putative SAOUHSC_02960

*sRNA names are given according to publication claim priority or most commonly used name. A list of previous analyses taken into account for this table is presented in Table S1. All sRNAs listed here were detected in at least 2 independent global expression studies performed in different strains. sRNA boundaries are given according to publications and GEO GSE104971 data. The presence of “UCCC” motif proposed by Geissmann et al. is indicated. NB: Northern blot; RT: RT-PCR; 5′- 3′: 5′ 3′ RACE*.

a *Mäder et al., 2016*.

b *Carroll et al., 2016*.

c *Geissmann et al., 2009*.

d *Abu-Qatouseh et al., 2010*.

e *Bohn et al., 2010*.

f *Lioliou et al., 2012*.

g *Pichon and Felden, 2005*.

h *Eyraud et al., 2014*.

i *Novick et al., 1993*.

j *Marchais et al., 2010*.

k *Beaume et al., 2010*.

**Table 2:** *Bona fide* sRNAs in HG003 with poor expression in the tested conditions of RNA-seq (this study) and tiling arrays (Mäder et al., 2016) datasets.

**Name**	**Other names**	**Start**	**End**	**Strand**	**UCCC**	**Validations**	**Comments**
Sau-27	srn_2690	1219192	1219282	+	–	NB^d^	No signal
Sau-85	srn_2760, SAOUHSCs165 (but longer)	1252254	1252305	+	–		Poor expression
RsaB	srn_3410, SAOUHSCs049, SAOUHSC_01844	1750160	1750216	+	+	RT^c^ 5′3′^c^	Poor expression; possibly 3'UTR of SAOUHSC_01844
sRNA334	srn_9480, SAOUHSCs242	2214760	2214889	+	–		Poor expression
sRNA390	srn_9510, SAOUHSCs250	2629688	2629848	+	–		Poor expression

*For explanations, see Table 1 legend*.

### Transcription factor binding sites in sRNA promoter regions

The 49 predictions of N315 Transcription Factors Binding Sites (TFBS) were downloaded (“reference regulons,” version 4.0, Fasta format) from the RegPrecise web site (Novichkov et al., 2013). Equivalences were searched for strain NCTC8325 as follows: When TFBS predictions are supported by only one promoter sequence in N315, we collected the predicted TFBS from other *Staphylococcus* species (using curl facilities of the RegPrecise web site). For each TFBS, a Position-Specific Scoring Matrix (PSSM) was computed with the MEME tool (Bailey and Elkan, 1994) using a background model built on the NCTC8325 genome sequence (fasta-get-markov in MEME suite, k-mer size of 3, -oops, -dna). The *S. aureus* NCTC8325 chromosome was scanned with the corresponding PSSM for each of the 49 TFBS with MAST (4.12.0) (Bailey and Gribskov, 1998). Only results on sRNA promoter regions (ranging from −100 nt to +50 nt from the 5′ sRNA end) and with a statistical *E-*value < 0.01 were conserved in order to report only the most probable predictions. However, this high stringency may discard effective TFBSs.

### sRNA coregulation and identification of putative sRNA targets

Genes coregulated with the sRNAs from Table 3 were identified using the web browser from Mäder et al. showing *S. aureus* expression data (Mäder et al., 2016). Relevant pages are indicated in Table S3. The RNApredator website (Eggenhofer et al., 2011) was used to predict sRNA-mRNA interactions between the sRNAs from Table 3 and the NCTC8325 genome (accession # NC_007795). In the absence of conservation data, RNAplex program used by RNA predator is among the best predictor (Pain et al., 2015). Results are presented in Data Sheet 2.

**Table 3:** List of transcription factor motifs found by MAST analysis (*E*-value < 0.01) in the putative promoter region (−100 to +50 nts from transcription start sites) of *bona fide* sRNAs.

**sRNA**	**TF**	**Effector(s)**	**TF function**	**References**
RNAIII	AgrA^*^ BirA SrrA^*^	Cell density (AIP) Biotin NO, anaerobiosis	Regulation of quorum sensing Biotin metabolism Anaerobic switch	Mäder et al., 2016; Novick and Geisinger, 2008; Yarwood et al., 2001
RsaB	Fur	Fe^2+^	Iron homeostasis	This study
RsaD	CodY	Branched-chain amino acids	Amino acid metabolism	Mäder et al., 2016
RsaE	SrrA^*^ Rex^*^	NO, anaerobiosis NAD	Anaerobic switch Anaerobic metabolism	Durand et al., 2015, 2017
RsaOG	CcpA	HPr, phosphocarrier protein; Fructose-1,6-diphosphate	Carbon catabolism	Mäder et al., 2016
RsaX20	Zur	Zn^2+^	Zinc homeostasis	Mäder et al., 2016
Sau-19	ArcR Rex	Arginine NAD	Arginine metabolism Anaerobic metabolism	This study This study
srn_2975	Fur NanR	Fe^2+^ N-acetylmannosamine-6-P	Iron homeostasis, Sialic acid catabolism	Mäder et al., 2016Mäder et al., 2016
sRNA207	BirA	Biotin	Biotin metabolism	This study
sRNA287	SarA^*^		Pathogenesis regulation	Mauro et al., 2016

Motifs were defined as given by Regprecise (Novichkov et al., 2013) for S. aureus N315 strain. We also search for GraRS, WalKR, and SrrA putative regulations using reported motifs (Sterba et al., 2003; Hartig and Jahn, 2012; Nicolas et al., 2012; Mäder et al., 2016; Durand et al., 2017);

* *corresponds to proposed or validated regulations reported elsewhere (discussed in the text) corresponding to motifs that did not pass the stringent E-value chosen or that are upstream of sequences selected for analysis. No DNA binding site was found for GraRS and WalKR*.

### sRNA conservation

sRNA sequence similarities were searched against a nucleotide database (see Table S2 for the list of strains). Complete genomic sequences were downloaded from the NCBI database. Similarity search parameters (blastall 2.2.26) were defined to report a maximum of hits (-e 1000 -W7) with specific scoring criteria (-r2 -G5 -E2) designed for sRNA identification (Ott et al., 2012). For each genome, only the blast hit with the best score was kept and divided by the score obtained in *S. aureus* NCTC8325. The resulting score ratios are represented by a color scale: the more the sequence of the hit is similar to the sRNA sequence, the darker the pixel is (R script). A 50% similarity ratio threshold was applied to define conserved sRNA genes.

## Results and hypothesis

### HG003 *bona fide* sRNAs

Based on a computational analysis of our HG003 RNA-seq data (GEO GSE104971), 88 UTRs, 22 antisense RNAs, 24 CDSs, 11 T-boxes, and riboswitches, and 3 toxin-antitoxin systems were annotated among the 527 putative regulatory RNAs found and indexed in the SRD database (Sassi et al., 2015). According to the definition given above, we considered a restricted list of 352 putative sRNA candidates to which we adjoined those of Carroll et al.'s and Mäder et al.'s sRNA lists. A gene-finding format (GFF) file including these putative sRNAs was generated (Data Sheet 1) and visually analyzed and compared to HG003 RNA-seq profiles using Artemis genome browser (Rutherford et al., 2000). In addition, HG001 tiling array profiles were scrutinized for each putative sRNA using the *S. aureus* expression data browser (http://genome.jouy.inra.fr/cgi-bin/aeb/index.py). From these inspections, we applied the *bona fide* criteria to compile a curated list of 41 *bona fide* sRNAs expressed in at least one biological condition in the HG003 strain (Table 1). We also added 5 *bona fide* sRNAs described in other strains but poorly expressed in HG003 and HG001 in the tested conditions. For instance, no expression was detected for Sau-27 in our HG003 RNA-seq or in HG001 tiling arrays data. As conditions might exist in which these sRNAs are expressed, we retained them in a separate table (Table 2).

**Figure 2:**
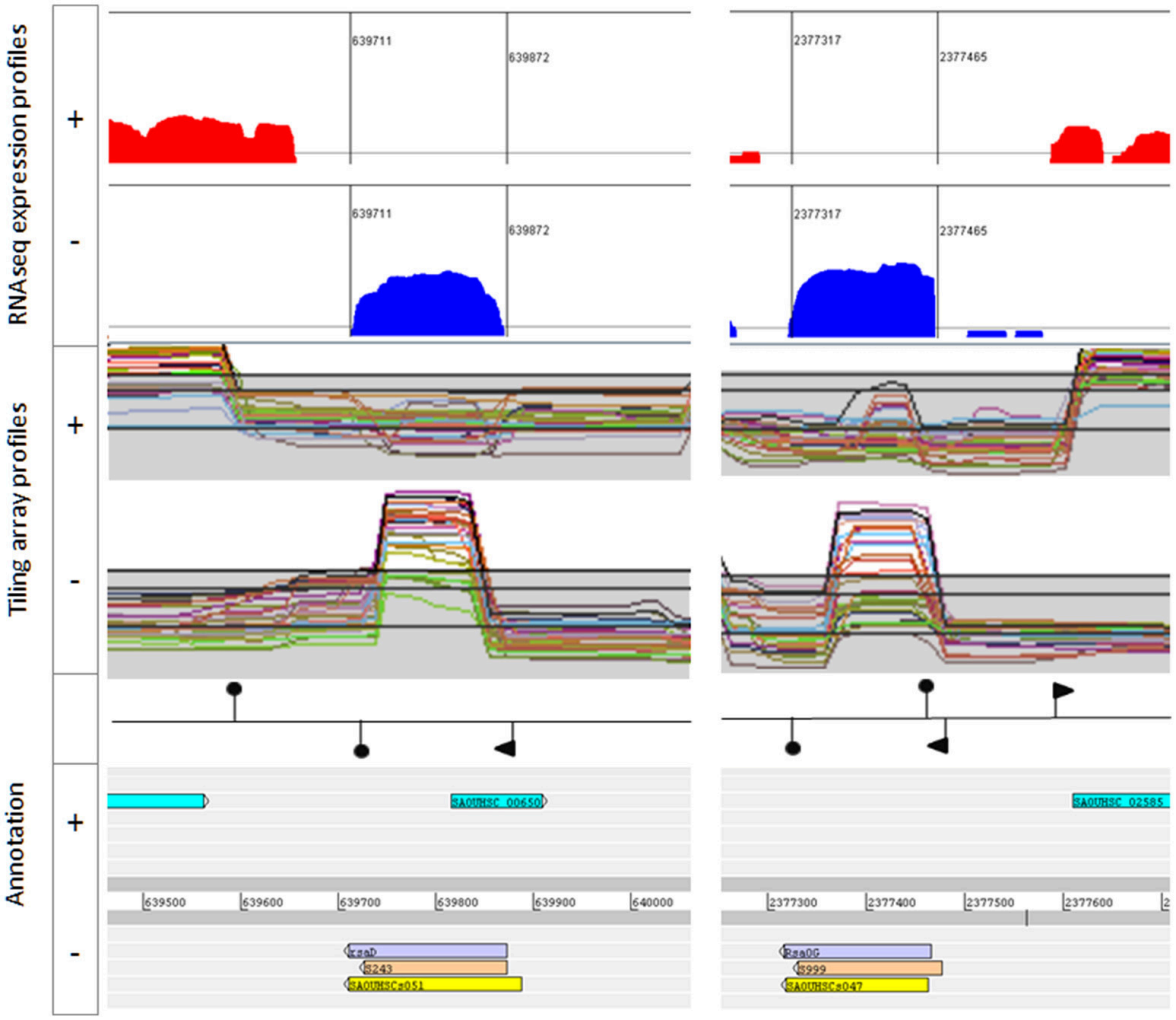
Example of *bona fide* sRNAs. RsaD **(Left)** and RsaOG **(Right)**. Upper: Artemis viewer window showing read log-coverages from pooled RNA samples extracted from HG003 grown in 16 growth conditions. Middle panel: screen snapshots of tiling array data from HG001 grown in different conditions (http://genome.jouy.inra.fr/cgi-bin/aeb/index.py, Mäder et al., 2016). Lower: annotations including genomic coordinates and sRNA names from Carroll et al. (yellow), Mäder et al. (light orange), and this study (mauve). Promoters (flags) and transcription terminators (hairpin loops) are placed according to Mäder et al. and/or TranstermHP software terminator predictions (Kingsford et al., 2007).

Most of the rejected sRNAs were found to be part of UTRs, or displayed a strong antisense-transcription signal. We discarded from the *bona fide* sRNA list, most RNAs with antisense expression and those likely part of type I toxin-anti-toxin systems [e.g., Teg13, RsaOI, srn_2335, SprC and S929 (Figure S1)]. However, we retained sRNA genes transcribed in antisense of putative small ORFs with no reported expression [e.g., Teg33, S596 and RsaD (Figure 2)]. Either the peptide does not exist or the antisense decay activity on the mRNA is efficient and completely turns off peptide expression.

Many short transcripts may encode small ORFs (sORF); however, for most of them, their expression is not confirmed. To avoid considering sORF genes as *bona fide* sRNA genes, we discarded those with either high conservation or with a hydrophobic domain. Four sRNAs (srn_0890, SprX2, RsaB and RsaX20) with putative sORFs were retained as their translation was uncertain. We also kept RNAIII expressing the delta hemolysin as its main function and structural part are associated with *trans*-acting regulation (Novick et al., 1993). Small peptides are also predicted by the microbial gene annotation platform MicroScope (Vallenet et al., 2017) for RsaA, Sau-76, 6S RNA, and sRNA264. Moreover, a ribosome profiling study suggests new ORFs corresponding to sRNA genes (e.g., RsaA_L_, SprB, 6S RNA, and tmRNA) (Davis et al., 2014); however, a ribosome binding on RNAs is not sufficient to confirm protein expression. In the absence of further biological validation and because sRNAs can have a regulatory activity both through RNA targeting and *via* the expression of small peptides, we retained all of them but their status may change in the future.

As the number and the depth of deep-sequencing analyses increase, separated adjacent sRNA transcription units can be merged. Here, we consider that Teg128 and Teg130 likely do not exist *per se* and annotation should be merged to correspond to RsaX20 (Figure S2). In another example, RsaA and Sau-76 share the same promoter, and RNase III-dependent processing generates shorter transcripts (Lioliou et al., 2012); in Table 1, we considered, as previously published, the two transcriptional entities, the short transcript RsaA, and the longer form RsaA_L_ (Figure S3).

The transcriptional study of HG001 in multiple growth conditions indicates a transcript named S390 downstream of the *rsaE* transcriptional terminator (Mäder et al., 2016). S390 has a putative terminator but no obvious promoter. Its expression is low compared to that of *rsaE*, possibly suggesting that S390 may result from a transcriptional terminator read-through of RsaE. Weak conservation of S390 beyond *S. aureus*, as opposed to high conservation of RsaE, questions its functional importance. RsaF is a 105 nucleotide sRNA. *rsaF* transcription was proposed to initiate from a promoter embedded in the *rsaE* gene, with expression resulting from transcriptional terminator read-through (Geissmann et al., 2009). As RsaF and its promoter were not detected in the transcriptome databases, we chose to consider just two transcripts, RsaE and the RsaE/S390 fusion.

Also, many previously reported sRNAs are now known to be part of UTRs. One example is Teg49: initially characterized as a *bona fide* sRNA (Beaume et al., 2010), it is also within the 5′UTR of *sarA* mRNA, yet Teg49 plays a *trans*-acting role by modulating *sarA* expression (Kim et al., 2014; Manna et al., 2017). For two recently proposed sRNAs, S1077 (Figure S4) and S736, which have their own terminators, authors showed that they are both part of longer transcripts that extend downstream of their terminators (Mäder et al., 2016) and are probably *cis*-acting elements. Alternatively, transcriptional terminator read-through from sRNA genes could generate longer regulatory RNAs.

The 4.5S RNA, which is the RNA component of the signal recognition particle ribonucleoprotein complex and is not a regulatory RNA was removed from the *bona fide* sRNA list. Two other sRNAs that interact with proteins, 6S RNA and tmRNA, were kept in the sRNA list as they may have regulatory functions (Makhlin et al., 2007; Cavanagh and Wassarman, 2014).

Our RNA-seq transcriptome data are similar to those produced by tiling arrays and presented in the *S. aureus* expression data browser (http://genome.jouy.inra.fr/cgi-bin/aeb/index.py). Indeed, many *bona fide* sRNAs listed in Tables 1, 2 are independently detected using these two methodologies. Our transcriptome analysis contains 27 *bona fide* sRNAs not annotated as such in the Mäder et al. study, although reported elsewhere. Six are located in repeat regions not evaluated by the tiling array method (e.g., S627 and S629 Figure S5). Others have either no expression or an expression level that does not fulfill the cut-off selection imposed by the authors (Figure S6). The slight expression differences observed [namely for, srn_0890, Teg147, Teg108 (Figure S6), Sau-85, RsaB, Sau-5949, SprB, Teg122, Teg124, sRNA334, Sau-19 (Figure S6), and sRNA390] could be due to allelic variation of the *tcaR* regulator between the two sister strains HG001 and HG003 or to specific expression of sRNAs in at least one of the conditions tested only in our dataset (e.g., heat shock).

### sRNA features

*S. aureus* is a low guanine-cytosine (33% GC) content member among Firmicutes. Local variation within the genome of this percentage may reflect DNA acquisition by horizontal transfer (Garcia-Vallve et al., 2000). This could be the case for Teg122, tmRNA, Teg147, srn_9345 and 6S RNA, whose GC content is above 40%. However, for tmRNA and 6S RNA, the composition is likely constrained by their interaction with proteins.

Base-pair associations between RNA molecules initiate with unpaired nucleotides; the pairing may then extend beyond these seed motifs. Using the MEME suite (Bailey et al., 2009), we searched for over-represented motifs within the 46 selected sRNAs, which may serve as seed of sRNA/RNA interactions. A conserved C-rich motif (UCCC) in unpaired regions was reported for several *S. aureus* non-coding RNAs (Geissmann et al., 2009). Impressively, this motif is present in 48% of HG003 *bona fide* sRNAs, often in multi-copy (from 1 to 5 motifs in HG003 RsaC; Tables 1, 2). sRNAs lacking this motif are often of small size. A stretch of C and/or G is possibly an efficient discriminating element since *S. aureus* is only 33% GC. As suggested by the authors, it also may indicate that sRNAs with GC-rich unpaired patches may share a mode of action (Geissmann et al., 2009). We have also looked for an alternative motif in sRNAs not featuring UCCC but found none, suggesting that each of these sRNAs would find their target with specific sequences.

### HG003 sRNA conservation

**Figure 3:**
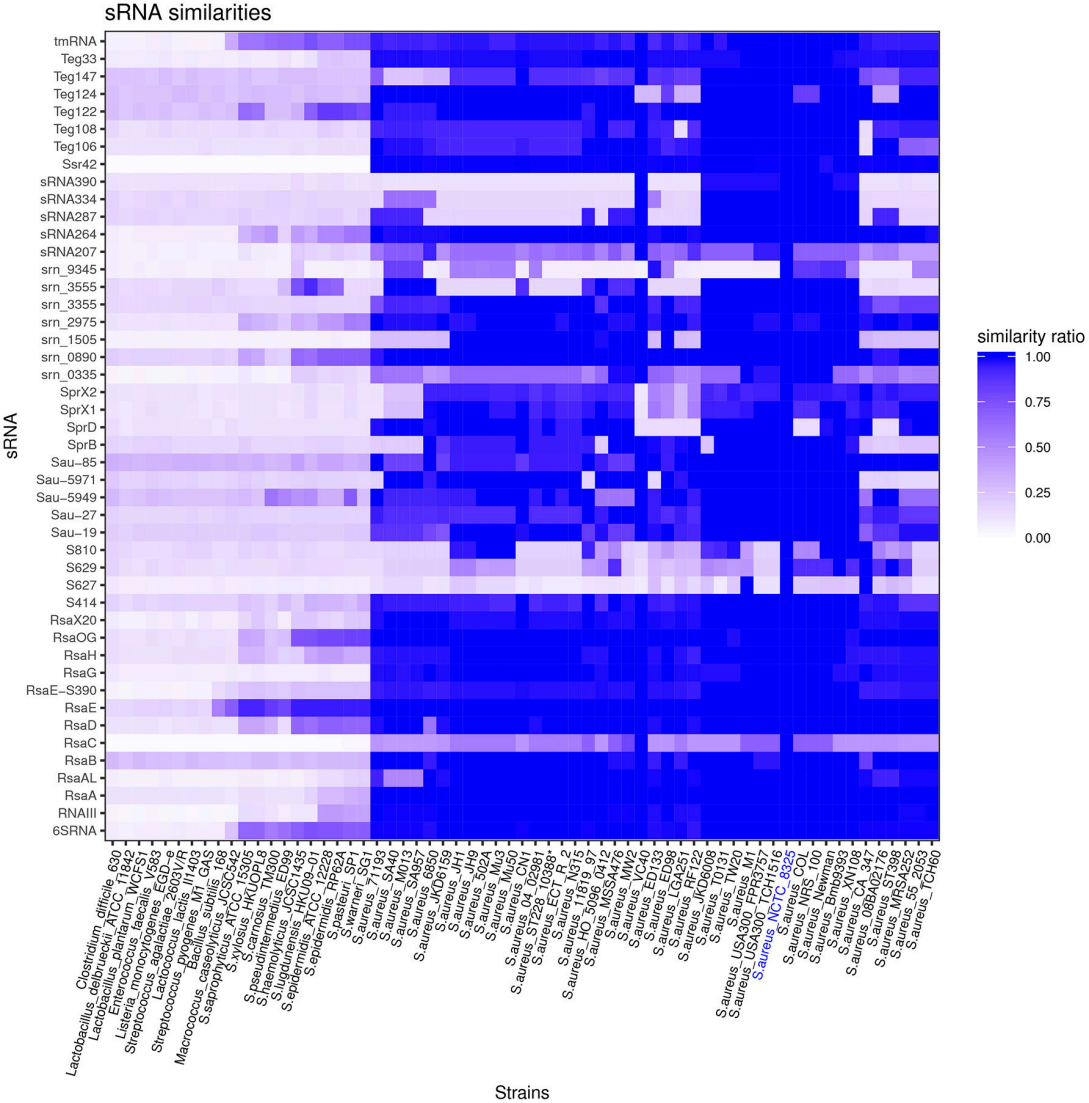
*Staphylococcus aureus* sRNAs conservation across the Firmicutes phylum. Vertical axe: list of *bona fide* sRNAs. Horizontal axe: list of *S. aureus* strains and other Firmicutes (for accession numbers, see Table S2). Similarity ratios between sRNAs of indicated strains and NCTC8325 sRNAs (reference strain) are represented by the indicated color code.

Among 46 HG003 *bona fide* sRNA genes, 54% are conserved in all tested *S. aureus* strains (Figure 3) and may be part of the core genome. 24% of the 46 *bona fide* sRNA genes are conserved among other species of the *Staphylococcaceae* family. However, most HG003 *bona fide* sRNAs are species specific. sRNA genes present on pathogenicity islands such as *sprX1, sprX2*, and *sprD* are *de facto* present solely in strains bearing these elements. *S629, S810*, and *srn_9345* genes are poorly conserved among the 43 *S. aureus* strains included in the analysis, and *S627* was found in only three of these strains, M1, CA347 and NCTC8325. Of note, *srn_3555*, while absent in many *aureus* strains, is conserved in non-*aureus* staphylococci such as *S. lugdunensis, S. haemolyticus*, and *S. epidermidis* suggesting its acquisition by horizontal transfer. The phylogenetic study suggests *rsaC* is poorly conserved in *S. aureus*. However, its conservation is probably underestimated due to the presence of repeat sequences, whose number varies according to strains (Figure S7).

Transfer-messenger RNA (tmRNA) and 6S RNA, which interact with SmpB protein and RNA polymerase, respectively, are widely conserved in bacteria. Apart from these sRNAs, RsaE is the only HG003 *bona fide* sRNA conserved in distantly-related Firmicutes. It differs from *Bacillus subtilis* RsaE almost exclusively by its terminator region. This unusual conservation reveals an unexpected selective pressure to preserve RsaE sequence integrity; we hypothesize that in addition to its numerous mRNA targets (Geissmann et al., 2009; Bohn et al., 2010), RsaE may interact with a protein constraining the RNA sequence to ensure it regulatory activity.

### HG003 sRNA transcriptional regulation

Transcription in *S. aureus* depends on four sigma (σ) factors: σ^A^, the primary σ factor responsible for the transcription of most genes, σ^B^, involved in the general stress response, σ^H^, implicated in the competence state but cryptic in NCTC8325 (Morikawa et al., 2012), and σ^S^, the extracytoplasmic function sigma factor (Burda et al., 2014).

In *S. aureus*, the σ^B^ regulon comprises about 249 coding genes expressed from 145 promoters (Bischoff et al., 2004; Mäder et al., 2016). The σ^B^ consensus recognition site was used to find small σ^B^ regulated genes called *sbr* (for σ^B^-dependent small RNA) (Nielsen et al., 2011). Three were found in several strains including SH1000, a NCTC8325 σ^B+^ derivative. However, *sbrA* and *sbrB* encode putative small basic peptides and are not regulatory RNA genes. The 3' end of *sbrC* overlaps with the 3′ end of *mntC*, that codes a metal binding lipoprotein, and their corresponding RNAs interact *in vitro*, indicating that SbrC should be categorized as an asRNA. σ^B^-mediated regulation has been proposed for RsaA, RsaF and RsaD, as their expression was enhanced in σ^B^ proficient strains, and a characteristic σ^B^ promoter was found upstream of *rsaA* (Geissmann et al., 2009). However, so far, σ^B^ regulation was confirmed only for *rsaA* and its derivative rsaA_L_ (including *sau-76*) (Mäder et al., 2016). No σ^B^ promoter was found upstream of *rsaD* despite activation of the σ^B^ regulon by several growth conditions (Mäder et al., 2016). Hence, only one out of 46 *bona fide* sRNAs, *rsaA*, appears to be transcribed by σ^B^. Remarkably, while often associated with adaptation and stress responses, almost all sRNAs have σ^A^ promoters. Since they are usually modulated by specific growth conditions, their expression likely relies on additional regulatory factors.

Transcription factors (TFs) bind specific DNA sites that can be detected by biocomputing tools when consensus sequences are already described. We performed such analyses for the *bona fide* sRNAs using MAST (Bailey et al., 2009) and predicted TF regulation for 8 sRNA genes (Table 3). The putative regulatory targets, and those previously reported, are discussed below.

RNAIII activates virulence genes either directly or indirectly at high *S. aureus* cell density. It is positively regulated by the quorum sensing regulator AgrA (Novick and Geisinger, 2008). AgrA also activates its own operon and *psm* (phenol-soluble modulins) genes that encode toxins (Queck et al., 2008), sometimes inadvertently annotated as sRNA genes. A putative BirA binding motif is detected upstream of the RNAIII gene (Mäder et al., 2016). BirA is a biotin-dependent repressor that downregulates genes implicated in biotin synthesis and transport (Henke and Cronan, 2016). In addition, RNAIII is reportedly repressed by SrrAB, a two-component system involved in aerobic to anaerobic adaptation and energy metabolism similar to *B. subtilis* ResDE (Yarwood et al., 2001). SrrAB-dependent RNAIII repression may result from a direct interaction of SrrA with the *agr* P3 promoter (Pragman et al., 2004).

SrrAB has an opposite effect on RsaE expression compared to that on RNAIII. The absence of SrrAB results in a drastic reduction of RsaE and an SrrA binding motif is detected 125 nucleotides upstream *rsaE* transcriptional start site (Durand et al., 2015). In *B. subtilis*, expression of *roxS*, the *rsaE* ortholog, is submitted to a double regulation by the activator ResDE, the SrrAB functional homolog, and the redox sensing repressor of anaerobic metabolism Rex (Pagels et al., 2010; Durand et al., 2017). As an identical Rex binding motif is also present within the RsaE promoter region, this double regulation is likely conserved for *rsaE* in *S. aureus*. RNAIII and RsaE would both exert a role in response to impaired respiration and indeed, in *B. subtilis* the absence of RoxS, results in the modulation of genes related to redox homeostasis (Durand et al., 2015).

In anaerobiosis, ArcR, a Crp/Fnr family transcriptional activator, stimulates arginine utilization as an energy source (Makhlin et al., 2007). We found that *sau-19*, an sRNA gene poorly expressed in conditions thus far tested, has ArcR and Rex binding motifs; these motifs resemble each other and concern the same sequence. Full activation of Sau-19 may need growth conditions in which Rex is inactive and ArcR is active, as observed for the arginine deiminase pathway (Makhlin et al., 2007).

*S. aureus* adapts to nutrient shifts with dedicated TFs. CcpA is a master regulator of carbon utilization in Gram-positive bacteria (Halsey et al., 2017). It binds to catabolite-response elements (*cre*) DNA sequences, and may act as an activator or a repressor. A *cre* box is detected within the promoter region of *rsaOG* (alias *rsaI*). RsaOG regulation by CcpA is supported by its coregulation with other CcpA regulated genes such as *lip, putA, fadXEDB*, and *rocA* (Mäder et al., 2016) (Table S3). This sRNA with a predicted pseudoknot (Marchais et al., 2010) is strongly modulated by growth conditions, and is increased in oxidative stress, during stationary phase and in human serum (Howden et al., 2013; Carroll et al., 2016). Fructose-1,6-bisphosphate is an allosteric effector of CcpA function (Schumacher et al., 2007). Interestingly, in addition to sugar transporters (i.e., SAOUHSC_02520, SAOUHSC_02815), the fructose-1,6-bisphosphate aldolase (SAOUHSC_02926) is a putative RsaOG target (Data Sheet 2), which in turn may contribute to CcpA regulation.

In *S. aureus*, CodY is a pleiotropic regulator affecting expression of numerous metabolic and virulence genes in response to branched amino acid and GTP availability (Geiger and Wolz, 2014; Waters et al., 2016). The presence of a CodY box in the promoter region of *rsaD* suggests that this sRNA belongs to its regulon. This proposal is strongly supported by the observation that *rsaD* is expressed in the same condition as CodY-regulated genes such as *SAOUHSC_00962, mtnE-ddh*, and *oppBCDFA* (Mäder et al., 2016) (Table S3).

Iron starvation is known to limit bacterial development during infection, but at the same time, an excess of iron generates deleterious reactive oxygen radicals. Consequently, intracellular iron homeostasis is tightly controlled and in many bacteria, the iron-sensing regulator Fur is involved. RhyB is an important Fur-regulated sRNA conserved in many Gram-negative bacteria that represses numerous genes and contributes to virulence (Oglesby-Sherrouse and Murphy, 2013). Iron-responsive sRNAs with a similar function are also present in Gram-positive bacteria (e.g., Gaballa et al., 2008). From the HG003 *bona fide* sRNA list, Fur boxes were detected in front of *rsaB* and *srn_2975* (S596), suggesting their implication in iron homeostasis. Regulation of *srn_*297*5* by iron is supported by (i) its co-expression with *isd* and *sbn* genes related to heme/hemin and iron uptake and utilization, respectively and (ii) predicted targets that are related to iron metabolism (Mäder et al., 2016). Srn_2975 would be the *S. aureus* functional ortholog of RhyB. Two NanR binding motifs are also found upstream of *srn_2975*. NanR is a repressor controlling sialic acid (N-acetylneuraminic acid) catabolism enzymes that may play an important role during growth in the host (Olson et al., 2013). Like iron, the metal ion zinc is essential. Zur, a zinc-sensing Fur-like protein (Lindsay and Foster, 2001) regulates zinc intracellular concentration. One sRNA gene, *rsaX20*, is preceded by a Zur binding motif, and interestingly RsaX20 is co-expressed with genes from the Zur regulon (Mäder et al., 2016) (Table S3). Consequently, RsaX20 is possibly associated with metal homeostasis.

sRNA regulation can be directly linked to virulence and pathogenicity factors. Besides the Agr system, as an example, SarA is a transcriptional factor belonging to the core genome, which is implicated in infectivity and biofilm formation. SarA represses sRNA287 and SprC, two sRNAs located on the same pathogenicity island (Mauro et al., 2016).

The sRNA regulators discussed here are associated with quorum sensing, aerobic to anaerobic transition, carbon source availability, metal metabolism or infectivity. All these processes crucial for virulence and survival within the host indicate that functional studies of *S. aureus* sRNAs are essential for understanding the global regulatory network governing bacterial pathogenicity.

### HG003 sRNA transcriptional termination

Bacterial transcription terminates either at secondary structures formed by nascent RNAs (intrinsic termination) (Ray-Soni et al., 2016) or *via* the activity of a termination factor such as Rho (Grylak-Mielnicka et al., 2016). Notably, while essential in several bacteria including *Escherichia coli*, Rho is dispensable in *S. aureus* (Washburn et al., 2001). Transcriptome data of HG001 *rho* in three different conditions is available (Mäder et al., 2016). Most intrinsic terminators are detected by bio-computing analysis. The presence of terminators within intergenic regions was initially used as an indication of the existence of sRNA genes (Wassarman et al., 2001) with the general belief that sRNA genes have Rho-independent terminators. Using TransTermHP with default parameters (http://transterm.cbcb.umd.edu) (Kingsford et al., 2007), intrinsic terminators were detected for 38 sRNAs among the 46 retained for HG003. By analyzing HG001 *rho* transcriptomic data for the nine sRNAs with no detected intrinsic terminator (Mäder et al., 2016), we conclude that they had no apparent Rho-dependent termination.

Several sRNAs have their own promoter but are also expressed because of a terminator read-through from upstream gene resulting in a longer RNA. In several cases, the expression of the sRNA gene and its upstream gene (or operon) is remarkably co-regulated (e.g., *rsaG, srn_1505*) suggesting that both genes are associated with the same function. In this case, the sRNA promoter would be present to boost sRNA expression.

## Conclusion

Most reported small transcripts correspond to UTRs and asRNAs. Our curated analysis led to a number of *bona fide* sRNAs in *S. aureus* that is smaller than what would be expected from the compilation of all sRNA studies. Even among this restricted list, the regulation, targets and functions of these sRNAs are still mostly unknown. Studying *bona fide* sRNA genes present the advantage that their deletion, in principal, has no polar effect on adjacent genes, thus facilitating genetic approaches to search for phenotypes (Le Lam et al., 2017). Putative proposed sRNA regulators (Table 3) are starting points to elucidate their function. Indeed, sRNAs often act as effectors of the transcription factors controlling their expression. They are the polishing regulators that would fine tune genetic regulation and refine bacterial adaptability. Described sRNAs are mostly negative regulators and often act as invertors of regulatory responses: induction of an sRNA by a given activator may lead to gene down-regulation. The same reasoning applies conversely for a repressed sRNA. For the above-discussed regulators, exploring genes repressed by inactivation of a repressor or induced by the absence of an activator is a good hint to discover sRNA-targets.

The number of *bona fide* sRNAs is lower than initially proposed, although we expect that new candidates will be added to this group. Publications based on high-throughput sequencing data indicate dense transcription with numerous so far uncharacterized transcripts that are putative regulatory elements. This pervasive transcription is hidden and probably not selected in a natural environment; and mutations such as *rnc* and *rho* are required to unmask it (Lasa et al., 2011; Lioliou et al., 2012; Mäder et al., 2016). Active *S. aureus* RNA processing generates numerous alternative RNA species (Lioliou et al., 2013; Bonnin and Bouloc, 2015) and many transcripts have long UTRs with a regulatory role demonstrated only in a few cases (e.g., de Los Mozos et al., 2013; Bouloc and Repoila, 2016). It is likely that besides *bona fide* sRNAs, *S. aureus* has a plethora of RNA-based regulations nesting within these non-translated RNAs.

## Author contributions

TR, PB, and CM: Conception, design of the work. WL, TR, and TL: Data acquisition. CT-N, PB, and CM: Data analysis. TR, PB, and CM: Drafting of the work and revision.

### Conflict of interest statement

The authors declare that the research was conducted in the absence of any commercial or financial relationships that could be construed as a potential conflict of interest.
