# Supplementary material for: Assessment of Bona Fide sRNAs in Staphylococcus aureus
Source: Front Microbiol. 2018 Feb 20;9:228. doi: 10.3389/fmicb.2018.00228 (PMC5826253; doi:10.3389/fmicb.2018.00228)
Supplement: Supplementary file 1 [file Presentation1.PDF]

Supplementary Data

Assessment of *bona fide* sRNAs in *Staphylococcus aureus*

Wenfeng Liu[a], Tatiana Rochat[b], Claire Toffano-Nioche[a], Thao Nguyen Le Lam[a], Philippe Bouloc[a] and Claire Morvan[a]

[a] Institute for Integrative Biology of the Cell (I2BC), CEA, CNRS, Université Paris-Sud, Université Paris-Saclay, 91198 Gif-sur-Yvette, France.

[b] VIM, INRA, Université Paris-Saclay, INRA Centre Jouy-en-Josas, France.

Supplementary Data

- **Table S1:** *S. aureus* sRNA global studies p2
- **Table S2:** Strains used to study the sRNA conservation across the Firmicute phylum p3
- **Table S3:** sRNA Web access to coregulation data from *S. aureus* Expression Data Browser from (Mader et al., 2016) p4
- **Figure S1:** Putative toxin-antitoxin systems excluded from the *bona fide* list p5
- **Figure S2:** Data compilation reduces the number of putative sRNAs p6
- **Figure S3:** Alternative sRNAs due to transcriptional termination read-through p7
- **Figure S4:** UTRs excluded from the *bona fide* sRNA list p8
- **Figure S5 & S6:** *Bona fide* sRNAs p9
- **Figure S7:** Expression profile of RsaC in HG003 p11
- **Parameters for the DETRPROK analysis** p12
- **References** p13

Table S1: *S. aureus* sRNA global studies

**Strain**	**Main data type**	**sRNA number**	**Reference**
Mu50	*in silico*	191	(Pichon and Felden, 2005)
UAMS-1 MSSA from osteolmyelitis	Affymetrix microarrays	126	(Anderson et al., 2006)
RN6390	*in silico*	110	(Geissmann et al., 2009)
N315	*in silico*	250	(Marchais et al., 2009)
Clinical isolates: A3878I A3878III	Cloning and sequencing	142	(Abu-Qatouseh et al., 2010)
N315	RNA-seq	30	(Bohn et al., 2010)
N315	RNA-seq	195	(Beaume et al., 2010)
JKD6008 ; JKD6009	RNA-seq	409	(Howden et al., 2013)
N315 ; Newman ; 18 *S. aureus* + 10 other Staphylococci	RNA-seq + *in silico*	575	(Sassi et al., 2015)
NCTC8325 ; USA300 ; MRSA252	RNA-seq	~300	(Carroll et al., 2016)
HG001	Tiling array	~300	(Mader et al., 2016)
HG003	RNA-seq	501	This study

Table S2: Strains used to study the sRNA conservation across the Firmicute phylum

Genebank	Strain	Genebank	Strain
CP001844.2	*S.aureus*_04_02981	AL009126.3	*Bacillus_subtilis*_168
CP003808.1	*S.aureus*_08BA02176	AM180355.1	*Clostridium_difficile*_630
CP003194.1	*S.aureus*_11819_97	AE016830.1	*Enterococcus_faecalis*_V583
CP007454.1	*S.aureus*_502A	CR954253.1	*Lactobacillus_delbrueckii*_ATCC_11842
CP002388.1	*S.aureus*_55_2053	AL935263.2	*Lactobacillus_plantarum*_WCFS1
CP006706.1	*S.aureus*_6850	AE005176.1	*Lactococcus_lactis*_Il1403
CP003045.1	*S.aureus*_71193	AL591824.1	*Listeria_monocytogenes*_EGD-e
CP005288.1	*S.aureus*_Bmb9393	AP009484.1	*Macrococcus_caseolyticus*_JCSC542
CP006044.1	*S.aureus*_CA_347	AM295250.1	*S.carnosus*_TM300
CP003979.1	*S.aureus*_CN1	AE015929.1	*S.epidermidis*_ATCC_12228
CP000046.1	*S.aureus*_COL	CP000029.1	*S.epidermidis*_RP62A
FR714927.1	*S.aureus*_ECT_R_2	AP006716.1	*S.haemolyticus*_JCSC1435
CP001996.1	*S.aureus*_ED133	CP001837.1	*S.lugdunensis*_HKU09-01
CP001781.1	*S.aureus*_ED98	CP004014.1	*S.pasteuri*_SP1
HE681097.1	*S.aureus*_HO_5096_0412	CP002478.1	*S.pseudintermedius*_ED99
CP000736.1	*S.aureus*_JH1	AP008934.1	*S.saprophyticus*_ATCC_15305
CP000703.1	*S.aureus*_JH9	AE009948.1	*Streptococcus_agalactiae*_2603V/R
CP002120.1	*S.aureus*_JKD6008	AE004092.2	*Streptococcus_pyogenes*_M1_GAS
CP002114.2	*S.aureus*_JKD6159	CP003668.1	*S.warneri*_SG1
FR821779.1	*S.aureus*_LGA251	CP007208.1	*S.xylosus*_HKUOPL8
CP003166.1	*S.aureus*_M013		
HF937103.1	*S.aureus*_M1		
BX571856.1	*S.aureus*_MRSA252		
BX571857.1	*S.aureus*_MSSA476		
AP009324.1	*S.aureus*_Mu3		
BA000017.4	*S.aureus*_Mu50		
BA000033.2	*S.aureus*_MW2		
BA000018.3	*S.aureus*_N315		
CP000253.1	*S.aureus*_NCTC_8325		
AP009351.1	*S.aureus*_Newman		
CP007539.1	*S.aureus*_NRS_100		
AJ938182.1	*S.aureus*_RF122		
CP003604.1	*S.aureus*_SA40		
CP003603.1	*S.aureus*_SA957		
HE579059.1	*S.aureus*_ST228_10388*		
AM990992.1	*S.aureus*_ST398		
CP002643.1	*S.aureus*_T0131		
CP002110.1	*S.aureus*_TCH60		
FN433596.1	*S.aureus*_TW20		
CP000255.1	*S.aureus*_USA300_FPR3757		
CP000730.1	*S.aureus*_USA300_TCH1516		
CP003033.1	*S.aureus*_VC40		
CP007447.1	*S.aureus*_XN108		

Table S3: sRNA Web access to corregulation data from *S. aureus* Expression Data Browser from (Mader et al., 2016)

**sRNA**	***S. aureus* Expression Data Browser link**
RNAIII	http://genome.jouy.inra.fr/cgi-bin/aeb/viewdetail.py?id=S871_2093169_2093503_-1
RsaB	http://genome.jouy.inra.fr/cgi-bin/aeb/viewdetail.py?id=NA_1750105_1750194_1
RsaD	http://genome.jouy.inra.fr/cgi-bin/aeb/viewdetail.py?id=S243_639727_639872_-1
RsaE	http://genome.jouy.inra.fr/cgi-bin/aeb/viewdetail.py?id=S389_911368_911465_1
RsaOG	http://genome.jouy.inra.fr/cgi-bin/aeb/viewdetail.py?id=S999_2377331_2377476_-1
RsaX20	http://genome.jouy.inra.fr/cgi-bin/aeb/viewdetail.py?id=S1052_2484599_2484726_1
Sau-19	None
srn_2975	http://genome.jouy.inra.fr/cgi-bin/aeb/viewdetail.py?id=S596_1362874_1363048_1
sRNA207	Internal repeat
sRNA287	http://genome.jouy.inra.fr/cgi-bin/aeb/viewdetail.py?id=S774_1863829_1863923_-1

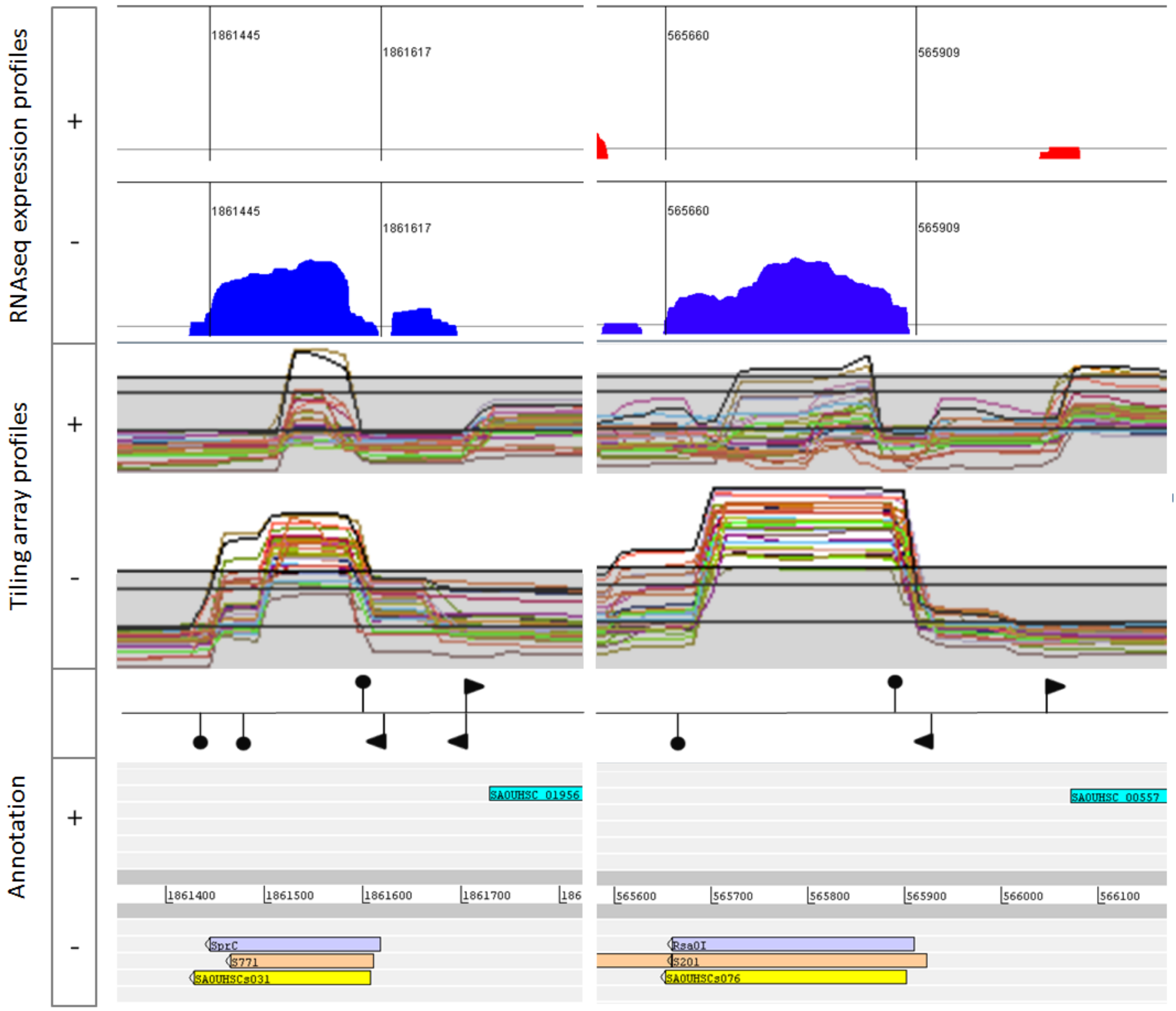

Figure S1: Putative toxin-antitoxin systems excluded from the *bona fide* list. SprC and RsaOI were excluded from the *bona fide* sRNA list because of high transcription levels on their opposite coding strand, in particular in RPMI and plasma (Mader et al., 2016).

Upper panel: Artemis viewer window showing read log-coverages from pooled RNA samples extracted from HG003 grown in 16 growth conditions. Middle panel: screen snapshots of tiling array data from HG001 grown in different conditions (http://genome.jouy.inra.fr/cgi-bin/aeb/index.py, Mäder *et al.* 2016). Lower panel: annotations including genomic coordinates and sRNA names from Carroll *et al.* (yellow), Mäder *et al.* (light orange) and this study (mauve). Promoters (flags) and transcription terminators (hairpin loops) are placed according to Mäder *et al.* and/or TranstermHP software terminator predictions (Kingsford et al., 2007).

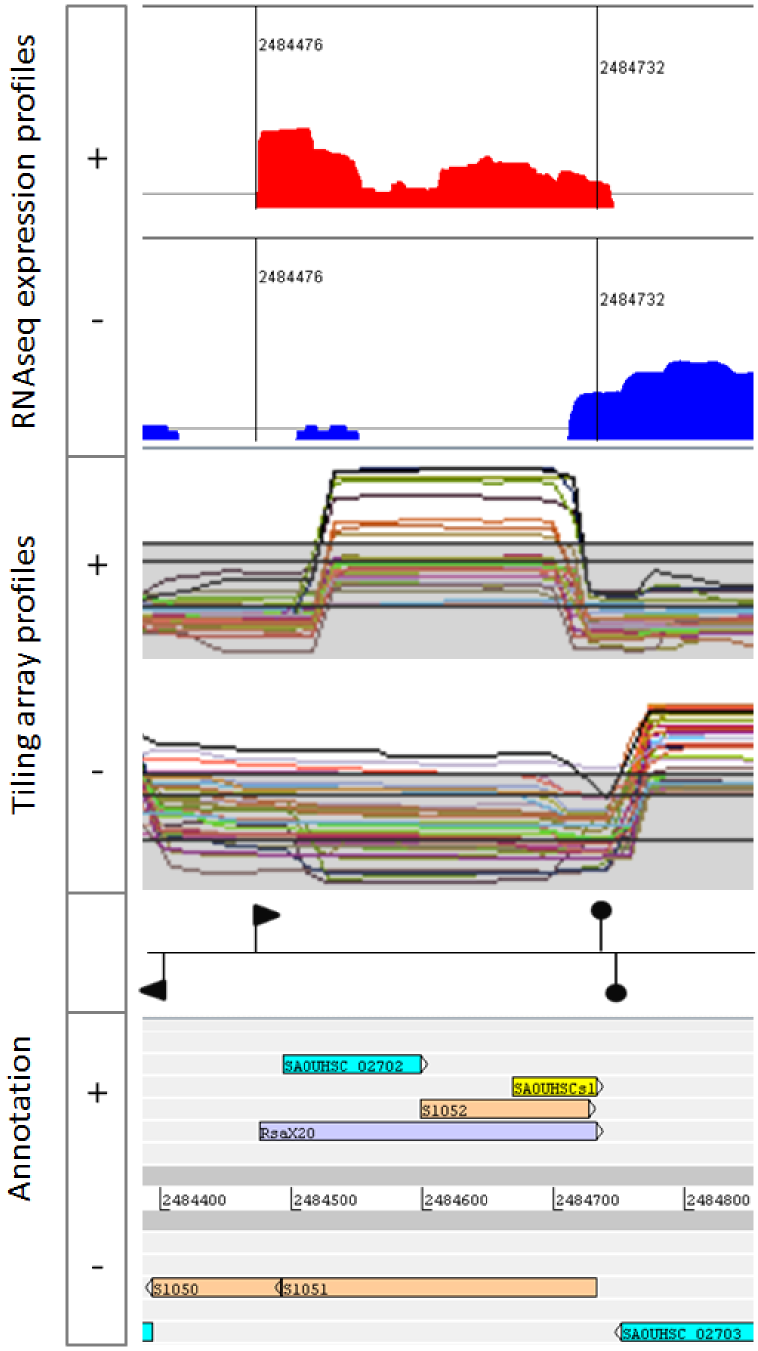

Figure S2: Data compilation reduces the number of putative sRNAs. In this example, Teg128 (SAOUHSC_02702) and Teg130 are considered as parts of RsaX20. For figure legend, see Figure S1.

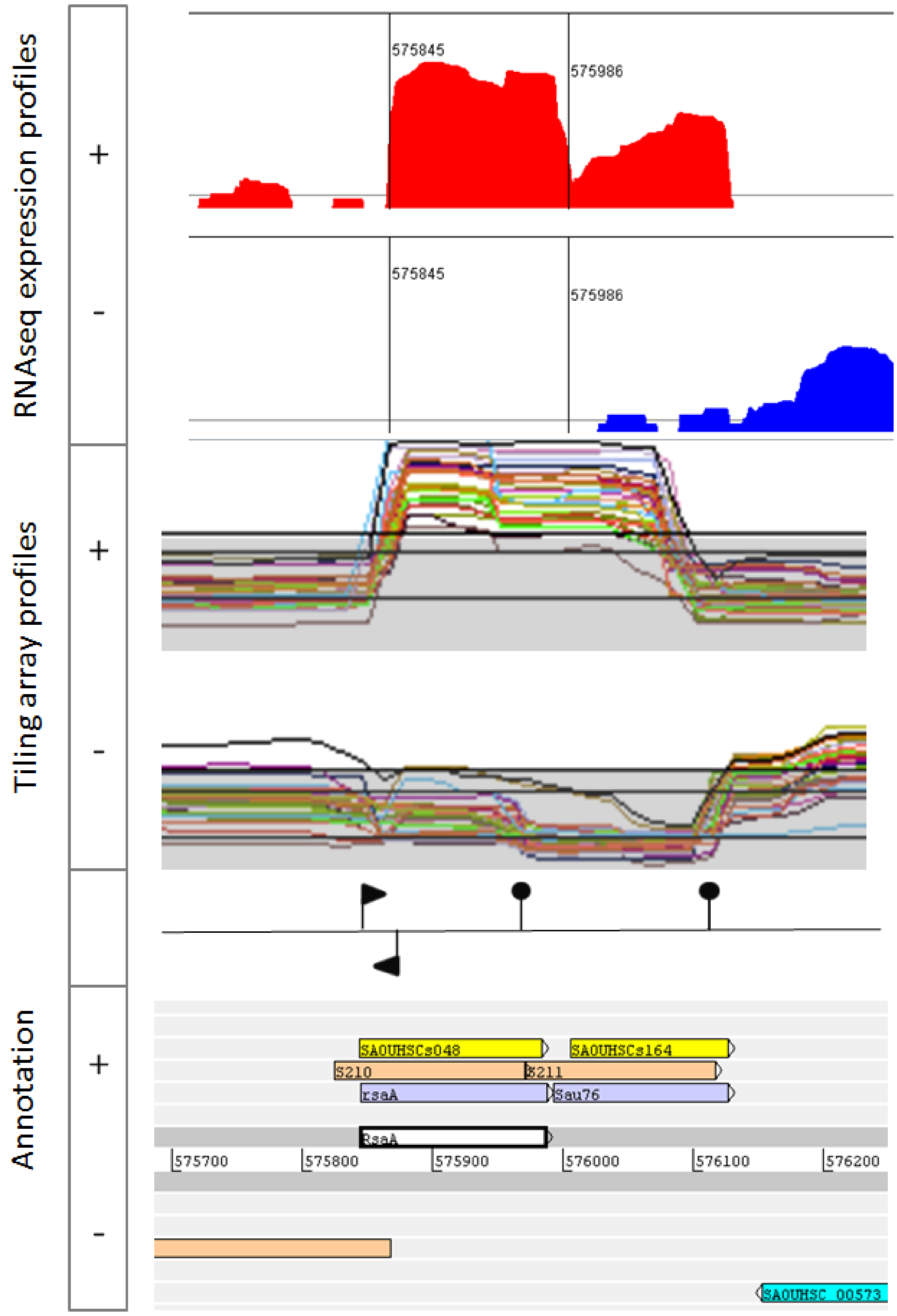

Figure S3: Alternative sRNAs due to transcriptional termination read-through. Transcription from the *rsaA* promoter leads to i) RsaA and ii) $RsaA_L$ after a transcriptional termination read-through. $RsaA_L$ includes Sau76. (see also (Lioliou et al., 2012)). For figure legend, see Figure S1.

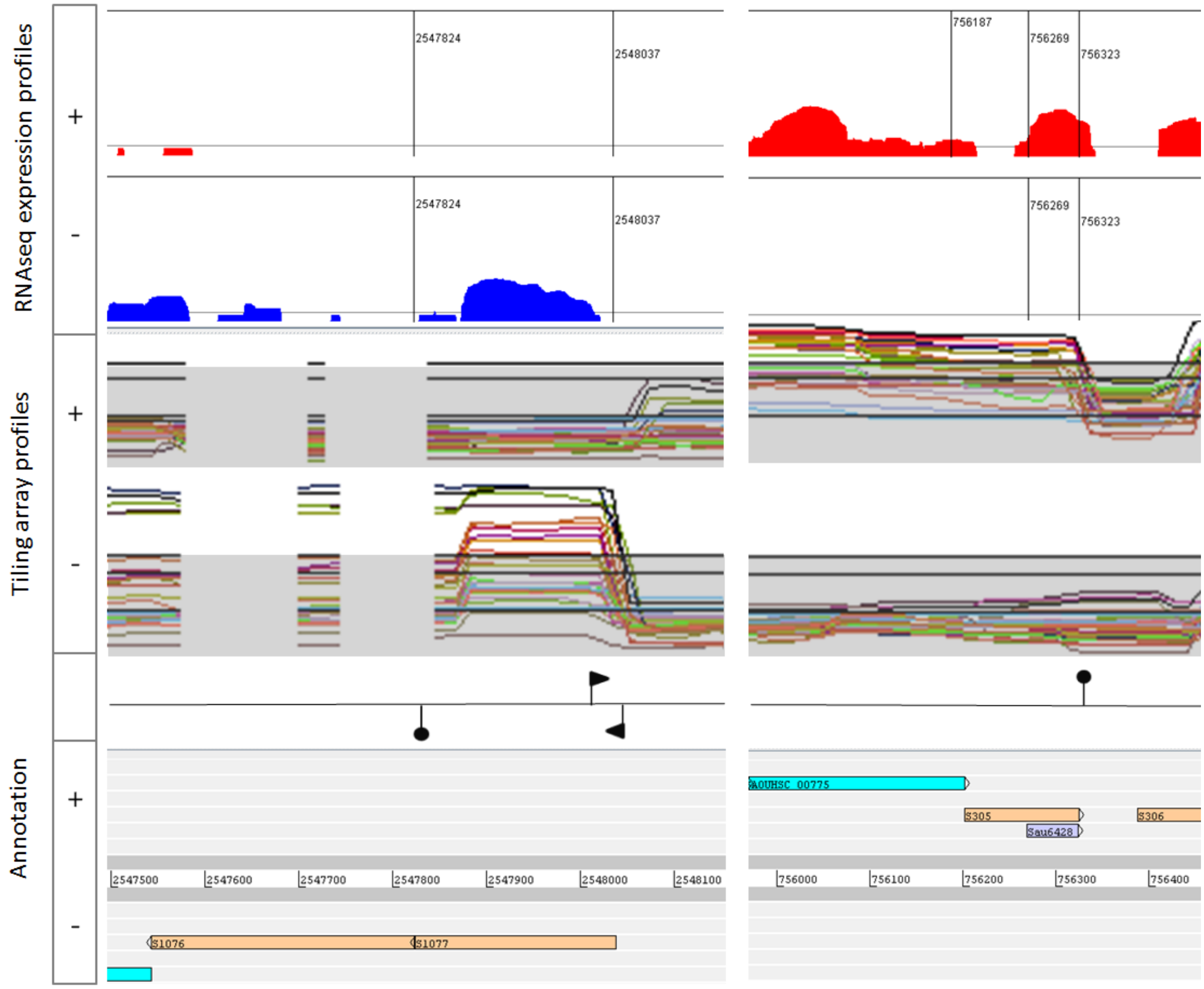

Figure S4: UTRs excluded from the *bona fide* sRNA list. A deep sequence coverage allows to conclude that S1077 is a 5'-UTR and may act as a *cis* regulatory element and that Sau6428, a former sRNA, is a 3'-UTR. For figure legend, see Figure S1.

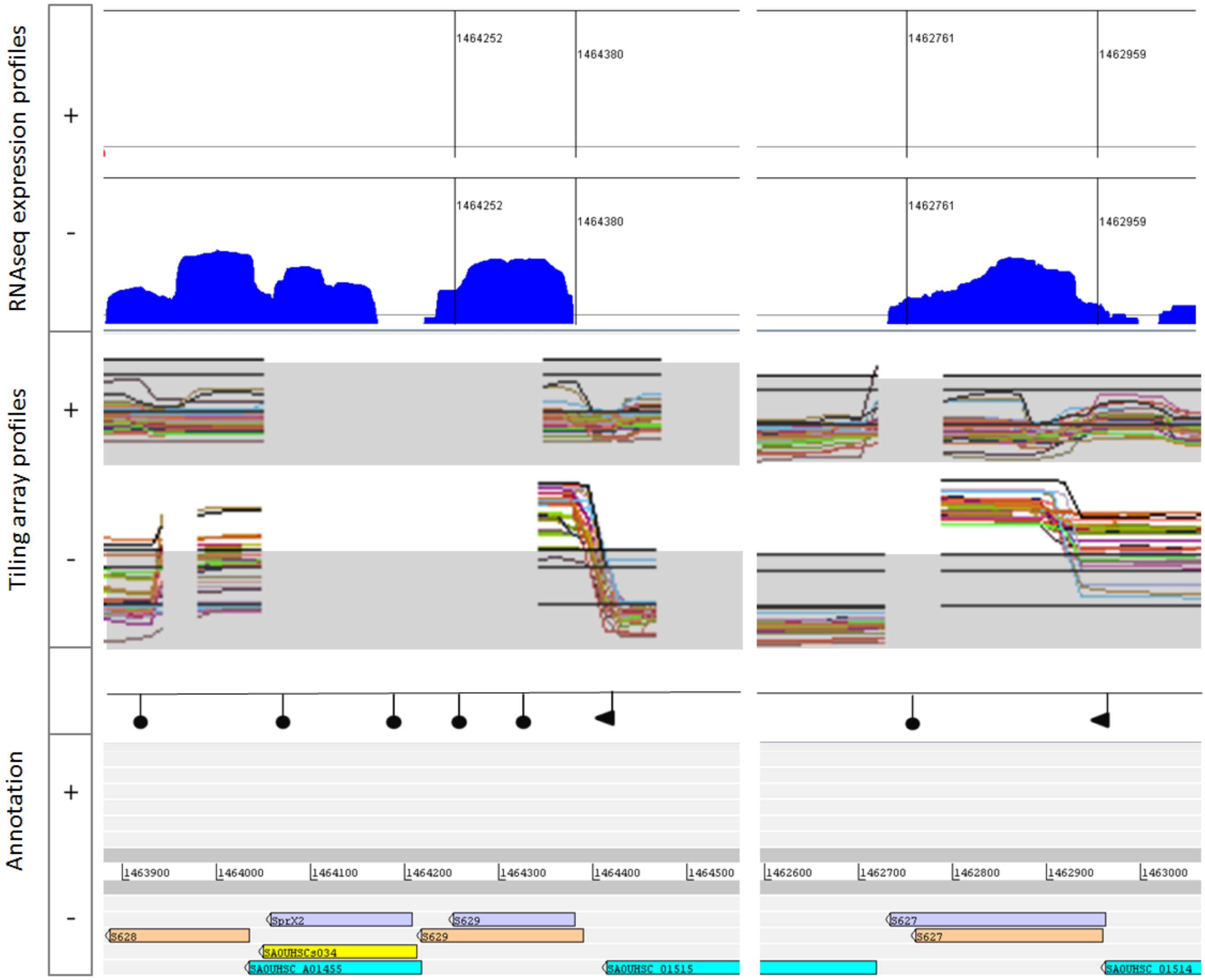

Figure S5: *Bona fide* sRNAs. Example of HG003 *bona fide* sRNAs, S627 and S629, not considered by Mader *et al.* study because of a partial sequence duplication (absence of read coverage in middle panel). For figure legend, see Figure S1.

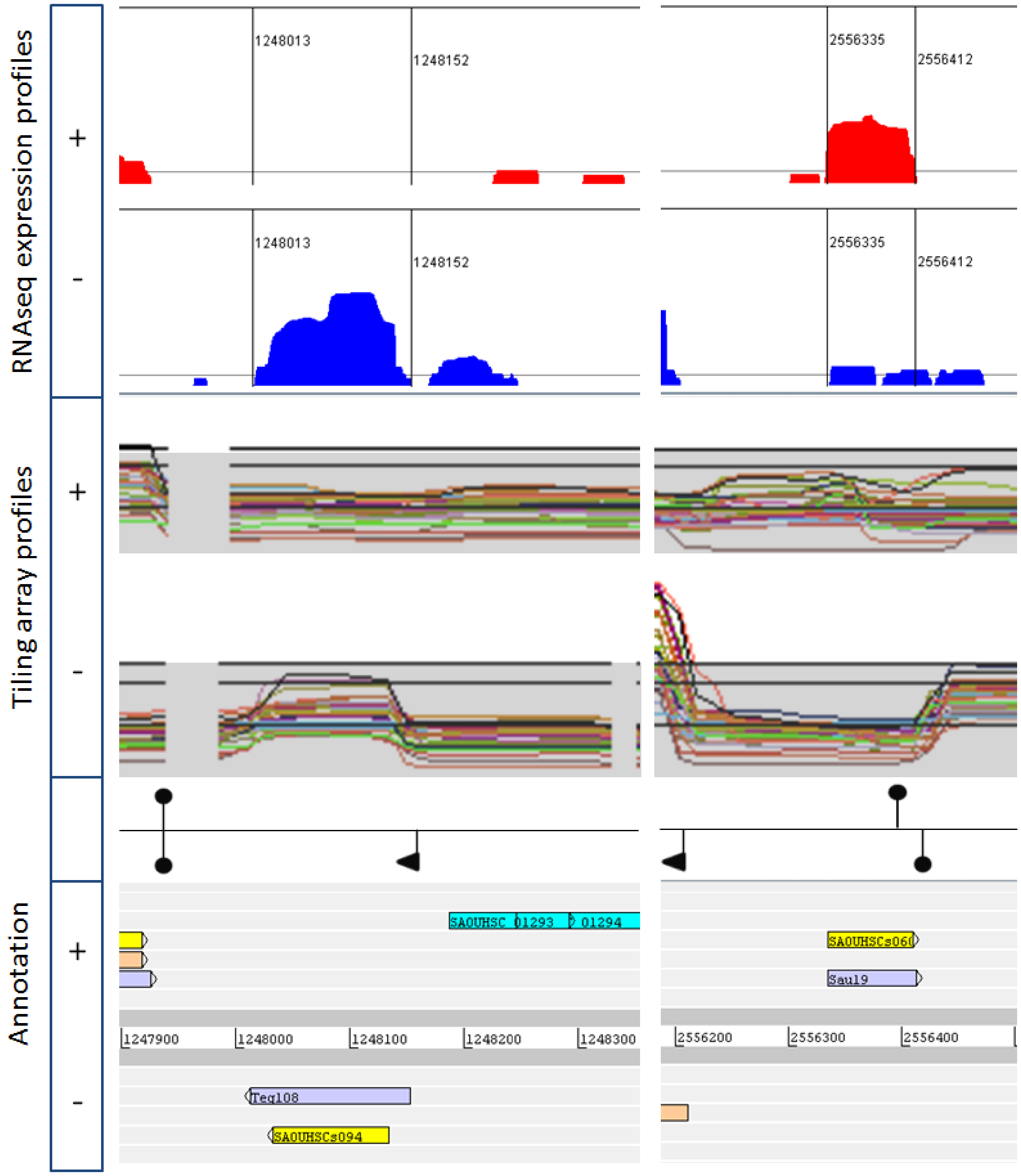

Figure S6: *Bona fide* sRNAs. Example of unfamiliar HG003 *bona fide* sRNAs because of low expression level in other analyses: Teg108 and Sau-19. For figure legend, see Figure S1.

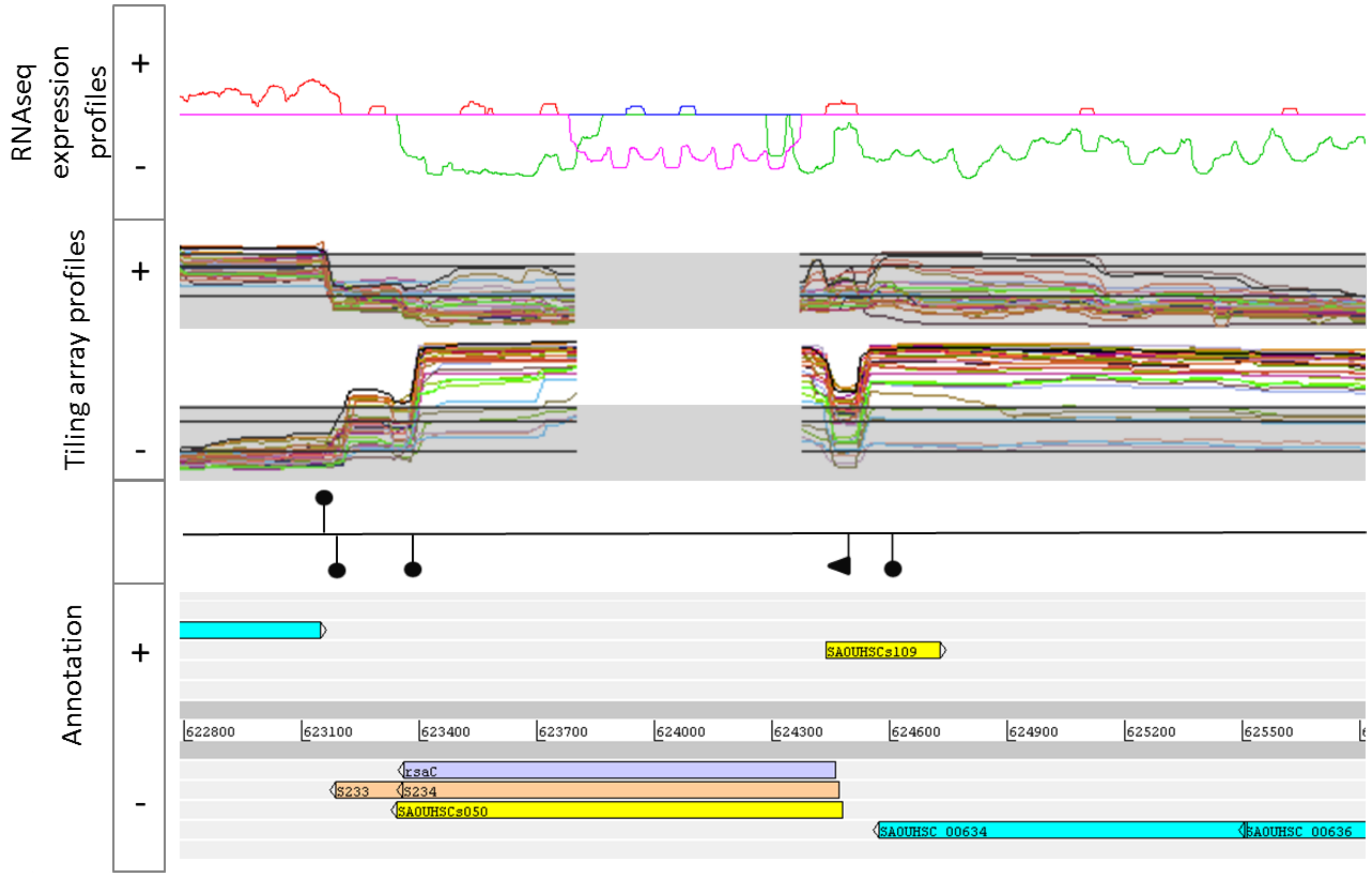

Figure S7: Expression profile of RsaC in HG003. Upper panel: Artemis viewer window showing read log-coverages from pooled RNA samples extracted from HG003 grown in 16 growth conditions. Red and blue lines for the + strand, green and pink lines for the minus strand. Red and green lines correspond to the mapping of reads with unique matches while blue and pink lines are reads with multiple matches spread over the genome map. As RsaC has internal repeats, this latter representation allows the visualization of RsaC expression. For middle and lower panels legend see Figure S1.

DETRPROK analysis:

Parameters used with DETRPROK_2.1.2.sh (Toffano-Nioche et al., 2012;Toffano-Nioche et al., 2013) on *Staphylococcus aureus* GENBANK CP000253.1 annotation without SAOUHSC_A.

-read_len 40
-features_list "CDS|rRNA|tRNA"
-op_gap 0
-clust_gap 0
-RNA_gap 20
-RNA_merge 50
-5utr_min_reads 10
-5utr_min_size 10
-5utr_coverage 0
-asRNA_min_reads 20
-asRNA_min_size 50
-asRNA_coverage 20
-sRNA_min_reads 10
-sRNA_min_size 50
-sRNA_coverage 4.4
-sRNA_inclusion 0.000000001
-all_feature false
-rm_tmp false
-verbose true

References

Abu-Qatouseh, L.F., Chinni, S.V., Seggewiss, J., Proctor, R.A., Brosius, J., Rozhdestvensky, T.S., Peters, G., Von Eiff, C., and Becker, K. (2010). Identification of differentially expressed small non-protein-coding RNAs in *Staphylococcus aureus* displaying both the normal and the small-colony variant phenotype. *Journal of Molecular Medicine-Jmm* 88**,** 565-575.

Anderson, K.L., Roberts, C., Disz, T., Vonstein, V., Hwang, K., Overbeek, R., Olson, P.D., Projan, S.J., and Dunman, P.M. (2006). Characterization of the *Staphylococcus aureus* heat shock, cold shock, stringent, and SOS responses and their effects on log-phase mRNA turnover. *J Bacteriol* 188**,** 6739-6756.

Beaume, M., Hernandez, D., Farinelli, L., Deluen, C., Linder, P., Gaspin, C., Romby, P., Schrenzel, J., and Francois, P. (2010). Cartography of Methicillin-Resistant *S. aureus* Transcripts: Detection, Orientation and Temporal Expression during Growth Phase and Stress Conditions. *PLoS One* 5.

Bohn, C., Rigoulay, C., Chabelskaya, S., Sharma, C.M., Marchais, A., Skorski, P., Borezee-Durant, E., Barbet, R., Jacquet, E., Jacq, A., Gautheret, D., Felden, B., Vogel, J., and Bouloc, P. (2010). Experimental discovery of small RNAs in *Staphylococcus aureus* reveals a riboregulator of central metabolism. *Nucleic Acids Res* 38**,** 6620-6636.

Carroll, R.K., Weiss, A., Broach, W.H., Wiemels, R.E., Mogen, A.B., Rice, K.C., and Shaw, L.N. (2016). Genome-wide Annotation, Identification, and Global Transcriptomic Analysis of Regulatory or Small RNA Gene Expression in *Staphylococcus aureus*. *MBio* 7.

Geissmann, T., Chevalier, C., Cros, M.J., Boisset, S., Fechter, P., Noirot, C., Schrenzel, J., Francois, P., Vandenesch, F., Gaspin, C., and Romby, P. (2009). A search for small noncoding RNAs in *Staphylococcus aureus* reveals a conserved sequence motif for regulation. *Nucleic Acids Res* 37**,** 7239-7257.

Howden, B.P., Beaume, M., Harrison, P.F., Hernandez, D., Schrenzel, J., Seemann, T., Francois, P., and Stinear, T.P. (2013). Analysis of the Small RNA Transcriptional Response in Multidrug-Resistant *Staphylococcus aureus* after Antimicrobial Exposure. *Antimicrob Agents Chemother* 57**,** 3864-3874.

Lioliou, E., Sharma, C.M., Caldelari, I., Helfer, A.C., Fechter, P., Vandenesch, F., Vogel, J., and Romby, P. (2012). Global regulatory functions of the *Staphylococcus aureus* endoribonuclease III in gene expression. *PLoS Genet* 8**,** e1002782.

Mader, U., Nicolas, P., Depke, M., Pane-Farre, J., Debarbouille, M., Van Der Kooi-Pol, M.M., Guerin, C., Derozier, S., Hiron, A., Jarmer, H., Leduc, A., Michalik, S., Reilman, E., Schaffer, M., Schmidt, F., Bessieres, P., Noirot, P., Hecker, M., Msadek, T., Volker, U., and Van Dijl, J.M. (2016). *Staphylococcus aureus* Transcriptome Architecture: From Laboratory to Infection-Mimicking Conditions. *PLoS Genet* 12.

Marchais, A., Naville, M., Bohn, C., Bouloc, P., and Gautheret, D. (2009). Single-pass classification of all noncoding sequences in a bacterial genome using phylogenetic profiles. *Genome Res* 19**,** 1084-1092.

Pichon, C., and Felden, B. (2005). Small RNA genes expressed from *Staphylococcus aureus* genomic and pathogenicity islands with specific expression among pathogenic strains. *Proceedings of the National Academy of Sciences of the United States of America* 102**,** 14249-14254.

Sassi, M., Augagneur, Y., Mauro, T., Ivain, L., Chabelskaya, S., Hallier, M., Sallou, O., and Felden, B. (2015). SRD: a Staphylococcus regulatory RNA database. *RNA* 21**,** 1005-1017.

Toffano-Nioche, C., Luo, Y., Kuchly, C., Wallon, C., Steinbach, D., Zytnicki, M., Jacq, A., and Gautheret, D. (2013). Detection of non-coding RNA in bacteria and archaea using the DETR'PROK Galaxy pipeline. *Methods*.

Toffano-Nioche, C., Nguyen, A.N., Kuchly, C., Ott, A., Gautheret, D., Bouloc, P., and Jacq, A. (2012). Transcriptomic profiling of the oyster pathogen *Vibrio splendidus* opens a window on the evolutionary dynamics of the small RNA repertoire in the Vibrio genus. *RNA* 18**,** 2201-2219.
